# Interventions to Improve Self-Efficacy in Colorectal Cancer Patients and/or Caregivers: A Systematic Review and Meta-Analysis

**DOI:** 10.1155/2021/4553613

**Published:** 2021-10-18

**Authors:** Jiali Gong, Caiping Hu, Meizhen Chen, Qian Cao, Qiuping Li

**Affiliations:** ^1^Wuxi School of Medicine, Jiangnan University, Wuxi, Jiangsu Province, China; ^2^Shanxi Provincial Cancer Hospital, Taiyuan, Shanxi Province, China

## Abstract

**Objective:**

High levels of self-efficacy (SE) in colorectal cancer (CRC) patients and/or caregivers enable patients to cope with cancer, reduce caregiver burden, and promote quality of life (QOL) in patients and caregivers alike. This review aims to (a) identify the SE theory sources covered by SE interventions or interventions, including targeting improved SE for CRC patients and/or caregivers, to guide future development of SE interventions; and (b) explore intervention effects based on SE theory through meta-analysis.

**Methods:**

Using five electronic databases—CINAHL, Cochrane Library, Embase, PsycINFO, and PubMed—a systematic search was performed in April 2021 to identify English or Chinese literature that studied improving SE interventions for CRC patients and/or caregivers. Manual screening of the articles' references list was also performed.

**Results:**

A total of 18 studies were found to be suitable and included in this review. Of the 18 studies that were included, 10 randomized controlled trials (RCTs) studies with 917 participants were eligible for meta-analysis. Interventions provide support for SE drawing on different sources of information. Performance accomplishment (PA) is the key source, with vicarious experience (VE) and verbal persuasion (VP) assisting in improving PA. Reducing negative emotional arousal (NEA) and improving positive emotional arousal (PEA) are also indispensable factors in improving SE. The meta-analysis results show that interventions based on the SE theory can bring about positive effects for CRC patients and/or caregivers.

**Conclusions:**

Different sources of information aimed at improving SE, covered by the interventions, including PA, VE, VP, NEA, and PEA, have been explored. Positive intervention outcomes that focused on improving SE for CRC patients and/or caregivers were identified and highlighted. For future SE interventions, we advocate choosing combination sources of SE information to design interventions. It is recommended that future SE improvement interventions should focus on improving PA, supplemented by increasing VE, while reducing NEA and providing useful VP.

## 1. Introduction

Colorectal cancer (CRC) is one of the most concerning and significant causes of death worldwide [1]. CRC treatments include surgery, chemotherapy, radiotherapy, and other biological immunological treatments [2]. With advancements in treatment approaches, the five-year survival rate among CRC patients has reached 65% (for colon cancer and rectal cancer, those figures are 67% and 64%, respectively) [3]. The early detection rate for CRC has increased, due to the promotion of cancer screening, together with the fact that patients are treated in time, leading to reduced mortality rates and an increase in the number of CRC survivors [4]. For cancer survivors, a key consideration for understanding the long-term effects of cancer and its treatment is quality of life (QOL) [5].

There is a growing recognition that the prerequisite for improving cancer patient QOL is to improve patient self-efficacy (SE) [6, 7]. SE, defined as a person's self-confidence in achieving behavioral goals, is an important component in Bandura's social cognitive theory, which is known as the SE theory, and plays an important role in the cancer coping process [8]. Evidence has shown that improved SE promotes behavioral changes and improves self-management ability, QOL, and confidence in coping with cancer [9]. Interventions to improve SE have been widely applied in cancer practice. For instance, one study, focusing on multimedia self-management intervention for lung cancer patients and their family caregivers, showed that both participant SE and QOL improved after the intervention [10]. Other reports have also pointed out that SE is an important determinant in improving QOL in cancer patients and family caregivers [11, 12]. A meta-analysis discovered that cancer coping SE is positively correlated with QOL [13].

Indeed, the evidence revealed that SE interventions tailored for cancer patients and/or caregivers resulted in many benefits [14–16]. One study found that SE pain intervention promoted cancer patient QOL [14]. Another study revealed that interventions enhancing the SE of lung cancer patients not only improved their ability to discharge respiratory secretions, enhanced their functional exercise capacity and exercise SE, but also reduced hospitalization costs. For lung cancer patients, SE interventions exerted a positive influence on psychosocial, physiological, and social factors [15]. The authors also recommended future studies to develop SE interventions and explore their long-term effects [15]. These SE interventions and findings fully demonstrate the importance of improving cancer patient and/or caregiver SE to improve their QOL.

Due to the mutual impacts seen between cancer patients and their family caregivers [17], improving SE is equally important for cancer patient caregivers [18, 19]. Through dyadic intervention for cancer patients and caregivers, Porter et al. found that dyadic intervention not only improved patient and caregiver SE but also reduced patients' psychological distress and pain and lessened caregivers' anxiety levels [18]. Gradually, more researchers are developing interventions to improve SE in cancer patients and/or caregivers [19].

According to reports, researchers have designed interventions based on Bandura's SE theory to improve cancer patient and/or caregiver SE, with positive results [20, 21]. Bandura's SE theory identifies four sources of SE, including performance accomplishment (PA), vicarious experience (VE), verbal persuasion (VP), and emotional arousal (EA), all of which contribute to SE development [22]. For cancer patients and/or caregivers, PA refers to a patient's successful experience in coping with cancer. Successful accumulated PA would be helpful in weakening the negative impact of occasional failures in coping with cancer. VE means that patients can be encouraged by observing others' positive behaviors in coping with cancer. VP involves providing advice to cancer patients and/or caregivers, encouraging them to believe they can successfully cope with difficulties and tasks, especially things that may have crushed them in the past. EA occurs when patients and/or caregivers are coping with cancer and includes both positive EA (PEA) and negative EA (NEA). PEA refers to the awakening of emotions, such as feeling proactive and optimistic, which sees patients more willing to actively cope with cancer and solve difficulties. On the contrary, NEA causes patients to exhibit more avoidance behaviors. Nevertheless, no specific review has been found to specifically identify the sources of SE theories covered by SE interventions, including targets to improve SE, or determined intervention effects and content, to guide the future development of SE interventions.

In addition, findings from a meta-analysis have indicated that interventions based on theory have a more significant effect on improving cancer patient QOL [23]. To date, some researchers have conducted randomized controlled trails (RCTs) to clarify the implementation effects of interventions based on SE theory [24, 25]. However, no related meta-analysis has been identified to explore the intervention effects, based on SE theory, on CRC patients and/or caregivers. To conduct such a meta-analysis of interventions based on the SE theory on CRC patients and/or caregivers and compare this with non-SE theory-based interventions would be beneficial to clarify the intervention effects and further delineate the effect of SE theoretical guidance.

In summary, this review was designed to (a) identify the SE theory sources covered by SE interventions or interventions, including targeting improved SE for CRC patients and/or caregivers, to guide the future development of SE interventions; and (b) explore intervention effects based on SE theory through meta-analysis.

## 2. Methods

### 2.1. Search Strategy

**Figure 1:**
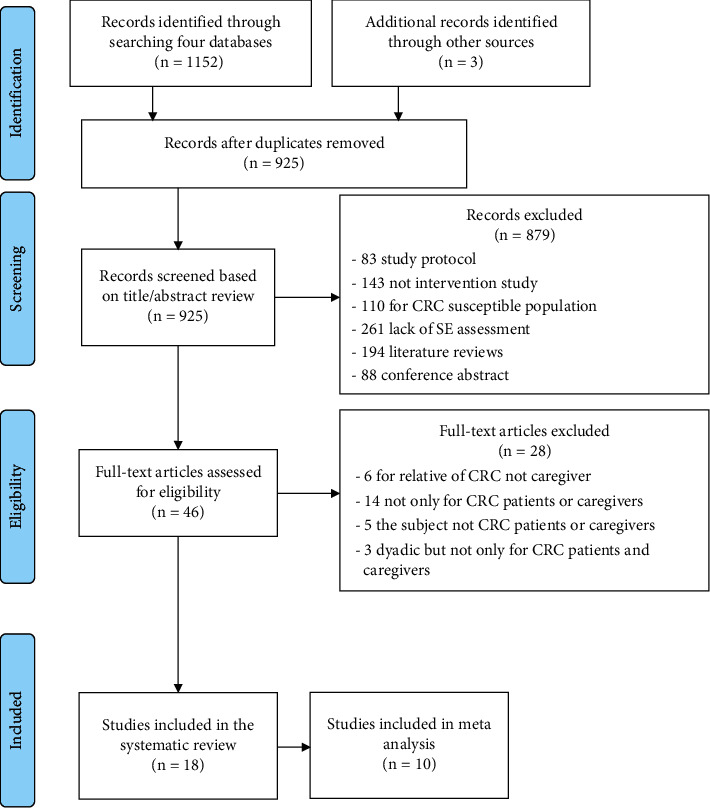
The flow diagram identifying the literature.

Literature focusing on interventions designed to improve the SE of CRC patients and/or caregivers was identified. Five databases, including CINAHL, Cochrane Library, Embase, PsycINFO, and PubMed, were used to identity articles from database inception to April 2021. The search terms were as follows: “colorectal neoplasm” or “colorectal cancer” or “colorectal tumor” or “colon cancer” or “colostomy” and “patient” or “survivor” or “caregiver” or “couple” or “spouse” and “intervention” or “program” or “programme” or “training” or “education” and “SE” or “social cognitive theory” or “bandura”. A manual screening of the articles' references list was also performed, naming “Other sources” in Figure 1. The detailed screening process is shown in Figure 1.

### 2.2. Inclusion and Exclusion Criteria

Articles satisfying the following criteria were included: (a) English or Chinese articles from the five databases' establishment to April 2021; (b) patients diagnosed with any stage of CRC and/or their family caregiver; (c) outcomes include SE measured using any SE scale; (d) intervention studies specifically conducted on CRC patients and/or their caregiver; (e) participants were adults (older than or equal to 18 years). The following articles were excluded: (a) commentaries, qualitative studies, literature review, dissertations, and conference abstracts; (b) did not measure SE (any aspect of SE, e.g., self-management efficacy, body-efficacy) as the outcome.

### 2.3. Data Extraction and Quality Assessment

Search results from different electronic databases were exported to EndNote (version X9), and duplicates were removed. The literature, study design, SE sources, intervention content, outcome measurements, and study outcomes were independently extracted by different examiners (see Tables S1 and S2).

The quality assessment was conducted independently by two reviewers using the criteria set out in the Effective Public Health Practices Project (EPHPP) [26] (Table 1). This assessment methodology has been proven to be effective and reliable, adapted to the effectiveness of systematic review interventions [26].

### 2.4. Meta-Analysis

**Figure 2:**
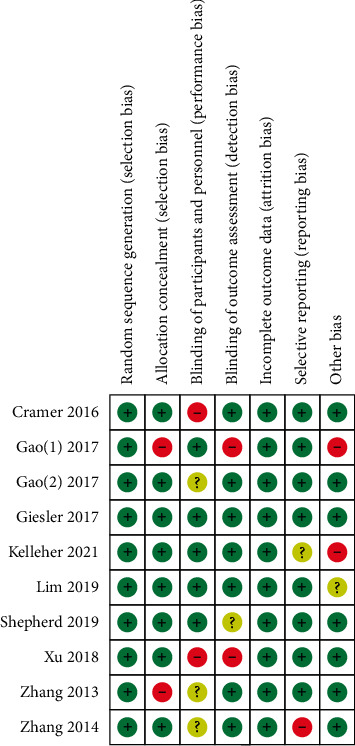
Summary of risk of bias.

We extracted the SE outcomes from RCTs based on the SE theory and from RCTs not based on SE theory, and a meta-analysis was conducted using the Cochrane Collaborations Review Manager 5.4 software. In RCTs, the control group received routine care or no intervention. The overall effect was calculated using mean and standard deviations (baseline and posttreatment values for the intervention and control groups) [43]. A random-effects model was used when moderate or high heterogeneity was detected, and a fixed-effects model was used when no or low heterogeneity was observed. If the same results for different studies were measured using different tools, and the resulting data were continuous data, then the standard average difference (SMD) was used. Statistical heterogeneity among studies was quantitatively examined using Cochran's *Q* test and the *I*^2^ statistic and visually using forest plots [44]. The heterogeneity levels were as follows: 0%–40%, without heterogeneity; 30%–60%, low heterogeneity; 50%–90%, moderate heterogeneity; and 75%–100%, substantial heterogeneity. When heterogeneity existed in the data, the possible causes were explored by sensitivity analysis [44]. RCT quality was assessed using the Cochrane Risk of Bias Tool [45] (see Figure 2). Publication bias was assessed using a funnel plot when more than 10 studies were available for SE outcome comparison.

## 3. Results

### 3.1. Study Selection

The literature search yielded a total of 1,152 potential eligible articles (Figure 1). We used EndNote X9 to exclude 230 duplicates and 879 irrelevant references through reading the titles and abstracts. Then, 28 studies were excluded by reading the full texts. A total of 18 publications were finally included in our analysis. The specific reasons for exclusion are presented in Figure 1.

### 3.2. Study Quality

Quality assessment was carried out for 18 articles. The quality rating of each study is presented in Table 1. Two studies received a “strong” rating, 13 studies a “moderate” rating, and the remaining three studies received a “weak” rating. Blinding was the most common reason for studies to be rated “weak” or “medium”. Although the quality rating is moderate, we did not exclude any literature with weak quality since this review is to extract interventions that assessed the SE of CRC patients. Obtaining an overview of the field is the main purpose, so we are not simply concerned with intervention effectiveness.

### 3.3. Intervention Characteristics

Table S1 summarizes the descriptions of each selected study and the sample characteristics. Among the 18 studies, 10 were conducted in Asia (China [24, 25, 31, 32, 34, 38, 42], Singapore [37, 41], and Korea [36]), six studies in Europe (the United Kingdom [27, 28, 40], Germany [29, 33], and the Netherlands [30]), while the other two studies were conducted in the United States. Study design included 12 RCTs, three pre-post design studies, two quasiexperimental studies, and one case report.

#### 3.3.1. Target Population

The number of participants across the 18 articles was 1,426 (ranging from 1 to 212), of whom 66.5% were male. Participants' mean age ranged from 44.47 to 68.7 years. Only two studies targeted CRC patients and their caregivers. Seven studies specifically reported patients' disease stages, including stages I–III [28, 29, 33] (*n* = 3), stages I–IV [39] (*n* = 1), stages II–IV [40] (*n* = 1), stages II–III [24] (*n* = 1), and advanced patients [41]. In addition, seven studies specifically reported patient treatment stages, including posttreatment [30, 31, 34, 35] and scheduled surgery or chemotherapy [24, 36, 37].

Eleven studies reported the attrition rate, which ranged from 3.7% to 41%. The reasons given by CRC patients or caregivers for declining to participate or withdrawing from the intervention were scheduling conflict [29, 38]; disease recurrence, too ill, death [38, 41]; no further treatment [24, 40]; change in hospital [38]; and chemotherapy [24, 36].

#### 3.3.2. Theoretical Framework

The interventions in the seven studies were designed based on the theoretical framework, while six studies [24, 25, 27, 35, 36, 42] developed interventions based on Bandura's SE theory, and one was based on both Bandura's SE theory and the stoma acceptance conceptual framework [37]. The SE theory is the core of Bandura's theory. Although different studies used different words to describe Bandura's theory, such as social learning theory [25] and social cognitive behavior theory [35], the core is that all used SE models. Two studies described how the theory was used [24, 25]. Researchers designed interventions based on the SE source and provided intervention support for patients encompassing four aspects: PA, VE, VP, and EA [24, 25].

#### 3.3.3. Intervention Duration

Fourteen studies reported the number of intervention course sessions, ranging from 1 to 10, with an average of 4.7 sessions. Nine studies reported the duration of each session. On average, face-to-face (F2F) sessions lasted longer than telephone sessions, with an average F2F session of 53.5 min (range: 60–120 min) and telephone contact of 36 min (range: 20–60 min). Sixteen studies reported the intervention period, ranging from 2 to 14 weeks, with an average time of 12 weeks. Sixteen studies reported the follow-up time, ranging from immediately after the intervention to 14 months, with an average follow-up time of three months. The number of follow-ups ranged from 1 to 5 times, with an average of two times.

#### 3.3.4. Intervention Delivery

Fifteen studies reported on the identity of the intervention deliverer, including nurse (CRC specialist nurse, general nurse) (*n* = 9, 60%), psychotherapist (*n* = 4, 26.7%), counselor (*n* = 1, 6.7%), and yoga instructor (*n* = 1, 6.7%).

Sixteen studies reported the intervention delivery format, including F2F (*n* = 9, 56.25%), telephone (referring to call) (*n* = 10, 62.5%), web-based (*n* = 6, 37.5%), booklet, or leaflet (*n* = 5, 31.25%). Most interventions used a combination of multiple delivery formats (two or more) (*n* = 9, 56.25%), and the most common combination was booklet, telephone, and F2F (*n* = 3, 33.3%).

#### 3.3.5. Self-Efficacy Intervention Content

In general, interventions that can improve SE mainly covered the following four sources of SE: PA, VE, VP, and EA.

*(1) Performance Accomplishments*. According to the concept of PA, we classified interventions focusing on skills training and knowledge strategies as PA. Skills training encompassed physical skills training (e.g., stoma nursing training [31, 37], coping with sexual challenges [39], yoga [29], walking [27, 35]) and psychological relaxation [24]. Knowledge strategies included physiological, psychological, and social knowledge. Physiological knowledge referred to cancer care (i.e., stoma care, cancer symptom management), helping cancer patients and caregivers cope with cancer together (i.e., sexual knowledge, communication, and mutual care) [35, 37, 38]. Psychological knowledge covered psychological adjustment and cognitive reconstruction [24, 35–38, 41, 42]. Social knowledge mainly referred to helping patients return to work (i.e., strategies for managing symptoms during work and skills for communicating with colleagues) [28].

*(2) Vicarious Experience.* VE means that patients learned from others' experiences solving a certain task and applied this knowledge skillfully. The VE content focuses on the physiological aspects and can be divided into health- and illness-related experiences. Health-related experiences included daily diet, rest, and exercise [33, 34]. Illness-related experiences mainly included coping with cancer symptoms and enterostomy care [33].

*(3) Verbal Persuasion.* VP aims to improve patients' SE through persuasion by others. VP content can be divided into three areas: physiology [24, 31, 34, 36–38, 40] (e.g., pro- and postoperative care, radiotherapy and chemotherapy care, symptom management), psychology [30] (e.g., clarify their feelings, cognitive-related processing), and society [28, 38, 39] (e.g., dealing with the relationship between work and cancer: regular work and rest, ensuring adequate water intake during work hours for ostomy patients to prevent dehydration, and for non-stoma patients, to avoid bending over; and communication tips when communicating with caregivers).

*(4) Emotional Arousal.* EA refers to changes in cognition, changing the way that patients deal with their relationship with the disease, arousing positive emotions to cope with the disease in a positive manner. The EA interventions were mainly designed to reduce NEA and increase PEA. The content includes managing negative emotions [41] (e.g., examples and discussions surrounding social roles and family impacts) and stimulating positive emotions (e.g., discussing problems F2F and relaxation practices, such as deep breathing and muscle relaxation [24]).

According to the differences in the four sources, different interventions can correspond to different SE sources: PA mainly covers skills training and knowledge education interventions, VE and VP mainly cover interventions that provide other patients' experiences or consulting services, and EA covers psychosocial interventions.

After the intervention content was classified according to the different SE sources, it was found that the content of SE's four aspects was not usually delivered to patients separately. For instance, most research to improve PA also provided VE/VP content to participants [24, 25, 28, 36, 37, 42]. Only two studies provided VE or VP separately. This is a reminder that future studies may need to support participants through SE's different aspects, and not just from one aspect (e.g., PA or VE).

In addition, it has been found that the intervention content corresponded to the participant's treatment or disease stage. For example, the PA content was given to participants who had already received initial treatment and had planned surgery or chemotherapy (e.g., self-management strategies [24], nursing strategies before and after a stoma operation [37]). Researchers also implemented pain and psychological distress coping skills training for patients with high pain scores [35]. Therefore, intervention content focusing on participants' diverse needs deserves to be considered and provided.

Further, although EA content can be classified into psychology, it is different from providing knowledge or advice to participants in terms of emotional regulation. It can be considered a specialized cognitive intervention therapy. Strictly speaking, it is impossible to simply classify the content of cognitive intervention therapy as psychological knowledge of PA, the psychological adjustment experience of VE, or the psychologically soothing suggestions of VP. EA emphasizes psychological internal adjustment and acceptance of the external environment (e.g., resolving anxiety and depression, improving acceptance of enterostomy) [25, 30, 41].

For dyadic interventions, the participants were mainly CRC patients and their spousal caregivers [38, 39]. PA focuses on enhancing the intimate relationship [39] (e.g., touching exercises for creative intimacy, devising new intimacy activities), effective and genuine communication [38], and teaching caregiver skills [38]. In addition to PA, VP assists in the mastery of skills. VP's unique feature is to directly provide suggestions to participants through an intervention implementer, who assists in the mastery of skills, such as communication frequency and preparation activities before couples' touching exercises.

### 3.4. Intervention Outcomes

Outcome measures are displayed in Table S2. We present the intervention outcomes in three areas according to this review's aims and SE association with QOL, namely, SE outcomes, QOL outcomes, and other outcomes.

#### 3.4.1. Self-Efficacy Outcomes

Eighteen studies used different scales to measure SE. The recognized SE scales that were used include the Body-Efficacy Expectation Scale (BEES) [29] (*n* = 1, 12.5%), the Chronic Disease Self-Efficacy Scales (CDSS) [28] (*n* = 1, 12.5%), the Cancer Behavior Inventory (CBI [41], CBI-B [38], CBI-B-D [33]) (*n* = 3, 16.67%), the Decision Self-Efficacy Scale (DSE) [40] (*n* = 1, 12.5%), the General Self-Efficacy Scale (GSES) [34] (*n* = 1, 12.5%), the Self-Efficacy Scale-28 (SE-28) [30] (*n* = 1, 12.5%), the Stoma Self-Efficacy Scale (SSES) [25, 31, 37, 42] (*n* = 4, 22.2%), the SE for Pain Management subscale of the Chronic Pain SE Scale [35] (*n* = 1, 12.5%), the Stanford Inventory of Cancer Patient Adjustment (SICPA) [24] (*n* = 1, 12.5%), and the Korean version of the Hospital Anxiety Depression Scale [36] (*n* = 1, 12.5%). In addition, self-designed questionnaires were designed to measure the SE of intimacy (enjoying intimacy, communicating effectively, dealing with one another effectively) [39] (*n* = 1, 12.5%), the SE of diet and physical activity [27] (*n* = 1, 12.5%), and general SE [32] (*n* = 1, 12.5%).

A total of 14 studies reported improved SE results. SE improvement in different areas is reported separately and can be roughly divided into two aspects: cancer-related SE [24, 28, 31, 32, 35, 38, 40–42] and general SE [27, 28, 30, 34, 36]. The results show that in cancer-related SE, improvements in stoma SE and pain SE, as well as improvements in decision-making and self-management SE, were of particular interest. In general SE, return-to-work SE [28] and diet and exercise SE [27] were emphasized. The effect sizes ranged from 0.08 to 2.41.

#### 3.4.2. Meta-Analysis

**Figure 3:**
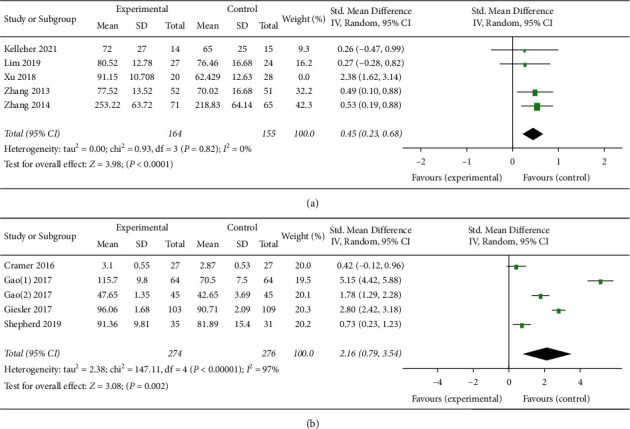
(a) Effect of interventions based on Bandura's self-efficacy theory. (b) Effect of interventions not based on Bandura's self-efficacy theory.

Of the 12 RCTs, five interventions were based on the SE theory, while seven interventions were not based on SE theory design. To more accurately understand the effects of interventions based on SE theory, this article will conduct a subgroup analysis of RCTs based on SE theory and RCTs not based on SE theory. Since two studies [39, 41] only provided the scores of the SE scale subitems, they could not be included in the meta-analysis. The remaining 10 RCTs were used to conduct a meta-analysis. Since the number of studies that were included did not exceed 10, funnel plots were not applied to measure publication bias. The results of the meta-analysis are shown in Figures 3(a) and 3(b), and the risk of biased assessment results is shown in Figure 2. The meta-analysis results are as follows.

A total of 367 patients were evaluated to examine the effect of the interventions based on Bandura's SE theory on the SE of CRC patients (see Figure 3(a)). The results showed high heterogeneity (*I*^*2*^ = 83%). In searching for possible sources of heterogeneity, we deleted the literature one by one and found that Xu et al.'s study might have been the source of heterogeneity. Figure 3(a) shows that interventions based on SE theory had significant effects after removing the study conducted by Xu et al. (*p* < 0.00001, 95% CI = 0.23–0.68, *I*^*2*^ = 0%).

When comparing the intervention effect between studies based on the SE theory and those not based on the SE theory, another meta-analysis was performed on the other five non-SE theory-based RCT intervention studies [29, 31–33, 40](*I*^*2*^ = 97%, *p*=0.002, 95% CI = 0.79–3.54) (see Figure 3(b)). No heterogeneity source was found in the sensitivity analysis. Comparative analysis showed that interventions based on SE theory (*p* < 0.00001) are more effective than those not based on SE theory interventions (*p*=0.002).

#### 3.4.3. Quality of Life

Eleven studies used different scales to measure QOL. The QOL scales that were used mainly included different versions of the European Organization for Research and Treatment of Cancer Quality of Life Questionnaires—such as EORTC QLQ-C30 V3.0 [42](*n* = 1, 10%), EORTC QLQ-CR29 [37] (*n* = 1, 10%), and EORTC QLQ-C30 + CR38 [30] (*n* = 1, 10%)—different versions of the Functional Assessment of Cancer Therapy Scales—such as FACT-C [29] (*n* = 1, 10%), FACT-G [35] (*n* = 1, 10%), FACT-Cv4 [36] (*n* = 1, 10%), and FACT-G (version 4) [24] (*n* = 1, 10%)—the medical outcomes study 12-item short form (SF-12) [38] (*n* = 1, 10%), the patient-generated quality-of-life questionnaires (PGI) [27] (*n* = 1, 10%), and the 36-Item Short Form Health Survey (SF-36) [34] (*n* = 1, 10%). In addition, there were self-designed questionnaires, designed to measure QOL [32].

Nine studies reported improvements in QOL, including overall QOL [27, 32, 35], and four aspects of overall QOL: physical well-being [30, 34, 38, 42], mental well-being [29, 30, 34, 36, 38, 42], social well-being [30, 34, 42], and functional well-being [34, 42].

Researchers found that overall QOL improved after the intervention [27] and at three months' [32, 35] and six months' follow-ups [32]. The effect sizes for these differences ranged from 0.40 to 3.63. Two studies reported that patients' QOL improved in all four aspects, with effect sizes ranging from 0.16 to 1.66 [34, 42]. Another two studies only reported improvements in mental well-being and produced moderate effects ranging from 0.57 to 0.66 [29, 36]. In addition, Döking et al. reported improvements in social well-being at four months' follow-up [30]. Luo et al. reported improvements in both physical and mental well-being in patients and caregivers alike. The effect sizes for these differences ranged from 0.18 to 0.33 [38].

#### 3.4.4. Other Outcomes (Bodily Function, Psychological Distress, and Dyadic Relationship)

*(1) Bodily Function*. There were 11 studies that reported physical health in terms of cancer symptoms (e.g., pain, sleep disturbance, fatigue) [24, 29, 30, 35], diet and nutrition [27], physical activity [27], patient ability (e.g., work ability, self-care ability) [25, 28, 31, 33, 34], and sexual function [39]. The effect sizes for these differences ranged from 0.18 to 1.76.

*(2) Psychological Distress*. This includes emotional distress [30, 39], anxiety and depression [24, 28, 29, 34, 36, 37, 40, 41], and negative emotions and positive emotions [38]. The effect sizes for these differences ranged from 0.05 to 0.90, and the mean effect size was 0.53, which represents a medium effect.

*(3) Dyadic Relationship*. Two of the dyadic intervention studies measured outcomes related to the dyadic relationship, such as dyadic communication [38, 39], intimacy [39], and relationship satisfaction [38]. These studies provided evidence to support that communication (*d* = 0.97) and intimacy (*d* = 0.51) may be improved through intervention for spousal caregivers [39], as well as communication and relationship satisfaction in patient (*d* = −0.18 to 0.16) and caregiver (*d* = −0.12 to 0.10) dyads [38]. However, there were also some inconsistent results: in terms of dyadic communication, one study reported positive effects for patients [38], while the other did not [39]. Collectively, the result of the dyadic relationship has a positive impact, with the effect sizes ranging from −0.12 to 0.97.

## 4. Discussion

This review explored existing interventions targeted to improve SE for CRC patients and/or caregivers. The positive outcomes are as follows: (1) our meta-analysis shows that the effects of SE theory-based interventions on SE may be more effective than interventions not based on theory and (2) efficacy in improving participants' QOL, and other outcomes (bodily function, psychological distress, and dyadic relationship). The four sources of SE theory may be the main reasons for the positive results.

The intervention content is mainly formulated by CRC characteristics and the SE theory source. Increasing participant PA has been valued by researchers, with many studies focused on improving PA (*n* = 12, 66.7%), which may confirm the results of previous studies: PA is the main source of SE [22]. However, a single PA intervention may not have a positive outcome. For example, the intervention effect of yoga training alone did not significantly improve SE [29], while the combination of both PA and VP or PA and VE has produced positive effects [24, 35, 37]. On the other hand, the effect of interventions only focusing on VE or VP may be weak. A study did not produce positive results by only providing patients with a website URL, where they could read about cancer coping skills to increase their SE [33]. Another research study also supports this hypothesis, with the study designing repeated consultation interventions to advise patients, but SE did not increase over time [40]. However, when faced with pressure and failure, any SE generated by suggestion can easily disappear. This does not mean that we should give up on trying to advise patients. Studies have shown that patients who received VP worked harder to complete the task than those who only had PA [22]. In summary, it is suggested that the main goal of future SE interventions should be to improve PA, while providing VE and VP to better assist the formation of PA.

Furthermore, the influence of EA on SE has been recognized. Studies have shown that anxiety and depression are negatively correlated with SE [46, 47]. When facing difficulties, the emotions that are awakened could be negative, such as anxiety and depression, or positive, such as optimism and positiveness, which determines the SE level. Cognitive behavioral therapy is a commonly used psychological intervention [48]. Comparing the two cognitive behavioral interventions involved in this review, the target populations are patients postenterostomy and patients with advanced CRC. In terms of content, for patients with enterostomy, the intervention focus is promoting patient acceptance of the stoma and reducing fear and depression, while for patients with advanced CRC, the focus is on symptom control and relaxation training. This suggests that we should provide interventions based on patient need at different disease stages.

The meta-analysis results showed that the intervention effects based on SE theory were significant. This is consistent with previous study results, and interventions based on theoretical design have proven their effectiveness [23]. Intervention studies based on SE theory provide patients with diversified services [24, 25, 36, 37, 42]. Some studies considered the four aspects of SE and provided support from these four aspects [24, 25]. Further, when compared with non-SE theory-based RCTs, it is found that these RCTs also have significant effects (*p*=0.002) but have heterogeneity (*I*^*2*^ = 97%), with differing measurement tools, intervention content, and delivery formats. Therefore, this result should be noted with caution. The main reason for the failure to conduct a meta-analysis on other outcome indicators, such as QOL, anxiety, and depression, is that some studies failed to report the above outcome indicators, or only used individual scale items for analysis. It is hoped that future research will use tested and completed scales for measurement.

SE theory has been widely applied in medicine and related aspects [24, 49]. Interventions guided by SE sources have shown positive outcomes in SE improvement for CRC patients [24, 25]. Our findings are also consistent with these previous studies in terms of improving SE targeting SE sources in CRC patients and/or caregivers, particularly, the combination sources of SE. Therefore, the authors assume that interventions designing combination sources of SE could potentially improve SE in CRC practice.

Nurses are the main intervention deliverers, and studies have shown that interventions provided by nurses bring more patient benefits [50]. More researchers are now using a combination of multiple delivery formats to implement interventions, and they highly value flexible delivery formats. When a F2F intervention is circumscribed by time and place, telephone or web-based interventions can play a role in transcending the limitations of time and space. The outcomes of this review show that whether F2F, telephone, or web-based, these interventions have produced a positive intervention effect. Future interventions can be tailored to select appropriate intervention delivery formats for different participants.

### 4.1. Study Gaps Identified

Seven of the 18 studies used theoretical frameworks, and all are SE theories. Although some studies used SE theory, most were not based on SE theory, nor did they describe in detail how the theory was used in the study. It is suggested that future SE interventions could be based on SE theory with clear explanations on how to use it.

There are various intervention measurements for SE and QOL. It is recommended that measurements for CRC patients and caregivers be unified, which will facilitate research statistics in future.

Only two of the studies that were included had implemented a dyadic intervention to improve the CRC patient and spousal caregiver intimate relationship and couples' ability to cope with cancer together. In the literature screening, we noticed the literature has reported that cancer patients and caregivers can work together to better cope with the problems caused by cancer [51–54]. The advantages of dyadic intervention have been clarified. Dyadic SE intervention can be designed in future for CRC patients and caregivers. Second, the dyadic intervention content focused on patient-caregiver communication, improved intimacy, and joint coping skills. It is limited to providing direct experience and suggestions and lacks VE and EA. For the future, we first advocate for the development of VE and EA content, and second, dyadic intervention should provide cancer care content.

Despite the fact that many RCTs explored the effects of improving SE intervention, the sample sizes were typically relatively small. In the future, a large RCT sample should be conducted to verify intervention effectiveness based on SE theory for patients with CRC.

### 4.2. Study Limitations

Several limitations in this review must be acknowledged. We were unable to perform a meta-analysis of all intervention studies due to differences in study design and quality, leading to the small sample size for the meta-analysis. In addition, this review is only limited to CRC, so interventions that targeted multiple cancers but included CRC have been excluded. Moreover, there are few interventions for caregivers or dyadic interventions for patients and caregivers, so we need to be cautious when interpreting the results and drawing conclusions for caregivers. Although these interventions exert a positive impact on SE, not all focused on improving SE as the main goal. In the future, more interventions focused on improving SE should be advocated for.

### 4.3. Recommendations for Future SE Intervention Research

We propose the following recommendations for future SE interventions, based on this exploration:

#### 4.3.1. Target Population

When the caregiver and patient cope with cancer together, the caregiver's SE is as important as the patient's. CRC patients-caregivers should be treated as a unit when providing interventions.

#### 4.3.2. Theoretical Framework and Intervention Methods

SE intervention using SE theory as a theoretical framework may have better outcomes: PA as the main source, VE and VP as auxiliary materials to improve PA. Reducing negative emotions and promoting positive emotions are also important.

#### 4.3.3. Intervention Type and Content

The specific content of the four SE aspects mainly revolves around physical, psychological, social, and dyadic coping with cancer: PA provides skill training and knowledge strategies; VE and VP content assists PA; and EA provides methods on how to decrease negative emotions and increase positive emotions.

#### 4.3.4. Intervention Duration

An appropriate intervention time (i.e., five sessions of 60 min (F2F)/30 min (telephone) each) with three months of follow-up is recommended.

#### 4.3.5. Intervention Delivery

More flexibility is important, without being restricted to one delivery format. In future, a combination of online and F2F delivery will be promoted. Trained professionals are the best candidates to implement interventions.

#### 4.3.6. Intervention Outcomes

Participants' SE, QOL, and other health outcomes, including body function, psychological distress, and dyadic relationship should be evaluated in the intervention outcomes.

### 4.4. Clinical Implications

The results of this review are of vital importance for clinical professionals who work with CRC patients and/or caregivers. First, this review gives a comprehensive introduction to the close relationship between interventions, SE, and corresponding mechanisms. To do so, efforts have been made in the following two aspects: (1) to clarify which parts of each intervention content were effective at improving SE, in terms of SE information sources based on Bandura's SE theory; and (2) to analyze the possible mechanisms by which each SE information source was effective at improving SE. Intervention content corresponding to SE sources may be more reasonable and scientific in developing interventions targeting SE improvement. Therefore, we advocate providing patients with knowledge and skills training in physiology, psychology, and society. Apart from direct teaching, we should also provide opportunities for communication between patients and pay attention to patients' negative emotions. It is necessary to deliver some F2F advice. Furthermore, our review demonstrates the effectiveness of interventions based on SE theory, so in the future, more comprehensive interventions should be designed based on SE theory.

## 5. Conclusion

This review explored existing interventions targeting improvements in SE for CRC patients and/or caregivers and explored the different sources of SE covered by the interventions. The meta-analysis results showed that interventions based on the SE theory have positive effects on CRC patients (*p* < 0.00001), and these effects are significantly higher than those of non-SE theory-based interventions (*p*=0.002). Although the meta-analysis results show that interventions based on both SE theory and non-SE theory are effective, non-SE theory interventions are highly heterogeneous and require careful consideration. Furthermore, we advocate choosing combination sources of SE when designing SE interventions in future. An important way to improve patient coping ability is to increase PA. Thus, the key goal of future interventions is to improve PA, which is supplemented to increase VE and VP, as well as helping to decrease NEA. This review also supports the idea of developing more dyadic interventions to improve dyadic SE in future to benefit dyadic coping with cancer.

## Figures and Tables

**Table 1:** Quality assessment of the included observational studies using the EPHPP tool.

Reference	Selection bias	Design	Confounders	Blinding	Data collection	Dropouts	Quality rating
Anderson et al. [27]	M	M	S	W	M	S	M
Bains et al. [28]	W	M	S	M	S	S	M
Cramer et al. [29]	M	S	M	M	M	M	M
Döking et al. [30]	W	W	M	M	S	S	M
Gao and Wu [31]	S	S	M	W	M	W	W
Gao et al. [32]	W	S	M	W	M	W	W
Giesler et al. [33]	S	S	M	W	S	S	M
Huang et al. [34]	S	S	S	M	S	M	S
Kelleher et al. [35]	M	S	M	M	S	M	M
Kim et al. [36]	S	M	S	W	S	S	M
Lim et al. [37]	M	S	M	W	M	S	M
Luo et al. [38]	M	M	M	M	S	S	M
Reese et al. [39]	W	M	M	S	S	M	M
Shepherd et al. [40]	M	S	S	M	S	W	M
Teo et al. [41]	M	S	M	W	M	M	M
Xu et al. [42]	M	S	S	W	S	W	W
Zhang et al. [25]	M	S	S	W	S	S	M
Zhang et al. [24]	S	S	M	M	S	S	S

*Selection Bias*. Strong: very likely to be representative of the target population and greater than 80% participation rate; moderate: somewhat likely to be representative of the target population and 60%–79% participation rate; weak: all other responses or not stated. *Design*. Strong: RCT and CCT; moderate: cohort analytic, case-control, cohort, or an interrupted time series; weak: all other designs or design not stated. *Confounders*. Strong: controlled for at least 80% of confounders; moderate: controlled for 60%–79% of confounders; weak: confounders not controlled for, or not stated. *Blinding*. Strong: blinding of outcome assessor and study participants to intervention status and/or research question; moderate: blinding of either outcome assessor or study participants; weak: outcome assessor and study participants are aware of intervention status and/or research question. *Data Collection Methods*. Strong: tools are valid and reliable; Moderate: tools are valid but reliability not described; Weak: no evidence of validity or reliability. *Withdrawals and Dropouts*: strong: follow-up rate of >80% of participants; moderate: follow-up rate of 60%–79% of participants; weak: follow-up rate of <60% of participants or withdrawals and dropouts not described. *Quality Rating*. S: strong; M: moderate; W: weak. Strong: if a study had no weak ratings and at least four strong ratings, then it would be considered strong. Moderate: if the study had fewer than four strong ratings and one weak rating, it would be rated moderate. Weak: if a study had two or more weak ratings, it would be considered weak.

## Data Availability

All data included in this study are available upon request by contacting the corresponding author.

## Supplementary Materials

Supplementary Materials Table S1: SE intervention characteristics; Table S2: SE intervention outcomes.
