# Supplementary material for: Interventions to Improve Self-Efficacy in Colorectal Cancer Patients and/or Caregivers: A Systematic Review and Meta-Analysis
Source: J Oncol. 2021 Oct 18;2021:4553613. doi: 10.1155/2021/4553613 (PMC8545593; doi:10.1155/2021/4553613)
Supplement: Supplementary Materials — Table S1: SE intervention characteristics; Table S2: SE intervention outcomes. [file 4553613.f1.doc]

Table S1. SE intervention characteristics

| **Author**  **Country**  **Reference number** | | **SD** | | **Target population (No. of participants, disease stage, Mean age, attrition %)** | | **Content of intervention**  **(intervention coverage domains^†^, intervention aim, intervention description)** | | **Dosage of intervention (No. of sections, time of each section, the duration of intervention, and the length of follow-up)** | | **Delivery of intervention (who, how)**  **/Theoretical framework** |
| --- | --- | --- | --- | --- | --- | --- | --- | --- | --- | --- |
| Anderson et al. (2010)  UK  [39] | | Feasibility study (pre-post design) | | N=28;  Any stage;  61.1 ± 9.0;  10% (suspected  recurrence) | | - PA  - intervention in overweight CRC patients to establish a healthy lifestyle, improve diet and exercise SE;  - Develop meeting plans (personalized physical activity and diet plan), set goals to assist with physical activity aids, and help identify high-risk situations (provide detailed feedback of accelerometer results and provide a pedometer and 4-week walking plan) and adjust goals after each meeting. | | 3;  1-2 hours of each section;  3 months;  Pretest, post-test. | | - Study counselor;  - F2F (one-to-one), telephone;  - **The Bandura’s SE theory.** |
| Bains et al. (2011)  UK  [40] | | Feasibility study (pre-post design) | | N=13;  Stage I-III;  56.25 ± 5.75;  41% | | - PA, VP  - Help patients return to work and adjust the relationship between disease and work;  - Return-to-work consultation discussed around with patient’s work content, treatment, feelings about continuing with or returning to work, illness and work management and adjustments; educational leaflet: managing symptoms at work, communication with employer, and work ability during and after treatment. | | 1;  Before intervention, 6 months follow-up. | | - Nurse (colorectal nurse specialists);  - F2F (one-to-one), written educational leaflet. |
| Cramer et al. (2016)  Germany  [42] | | RCT | | N=54;  Stages I-III;  68.26 ± 9.69;  5.2% (scheduling problem) | | - PA  - Improve patient's health-related quality of life;  - Yoga practice include activate or relax the body and mind, positively influence the intestinal organs, which gradually increase the intensity; yogic meditation techniques including mantra meditation and yoga nidra; each class ended with yogic breathing techniques; encourage practice at home. | | 10;  90-minute weekly;  10 weeks;  One week, 10 week, 22 week after intervention. | | - Yoga instructors;  - Web-based, F2F. |
| Döking et al. (2021)  Netherlands  [43] | | A case report | | N=1;  After permanent colostomy;  74 | | - VP, EA  - The primary aim was to reduce the distress-associated physical consequences of CRC and to improve mood and QOL;  - Content of intervention can divided into three treatment modules: (a) distress due to physical consequences, (b) depressive mood, and (c) anxiety and fear of cancer recurrence. | | 8;  90-minute weekly;  14 weeks;  Baseline (T1),  4 (T2), 7 (T3), and 14 months (T4) after intervention. | | - Psychotherapists;  - F2F, web-based, telephone |
| Gao et al. (2017)  China  [30] | | RCT | | N=128;  Rectal cancer after permanent colostomy;  54.1 ± 6.0; | | - VE, VP  - Using Cluster-based continuity nursing Intervention to promote patients' SE and self-care ability;  - Organize patient association and booklet to spread knowledge of disease to participants; ask patients about stoma-related knowledge through the telephone, understand the patient's existing problems in, and answer them in time. | | 4;  6 months;  One week, one month, 3 months, 6 months after discharge. | | - Nurse (stoma specialist nurse) or stoma therapist;  - Booklet, telephone, F2F. |
| Gao et al. (2017)  China  [34] | | RCT | | N=90;  Any stage;  IG: 44.47 ± 5.66  CG: 46.47 ± 5.45 | | - PA  - Explore the impact of self-care intervention on the QOL of patients after surgery;  - Strengthen patient confidence and teach self-care knowledge; improve stoma SE, learn the steps of stoma care and develop an action plan, maintain healthy behavior. | | 6 months  One , 3 and 6 months after discharge. | | - Nurse;  - Booklet. |
| Giesler et al. (2017)  Germany  [41] | RCT | | N=212;  Stages I-III;  54 ± 11.1;  5.78% (declined to participate) | | - VE  - The aim is to evaluate the CRC module of the website with regard to SE for coping with cancer and patient competence;  - The website displays information about health and illness collected and analyzed by the system. | | 6 weeks of access to the website;  6 weeks;  Baseline (T1), 2 weeks (T2) and 6 weeks (T3) during the visit to the website. | | - No specific report;  - Web-based. | |
| Huang et al. (2021)  China  [33] | Pilot study (quasi-experimental design and comparative study) | | N=119;  Rectal cancer patients with permanent colostomy;  IG: 45.82 ± 7.02  CG: 46.18 ±8.12 | | - VE, VP  - explore and analyze the effects of online training based continuous nursing care on the health-related QOL and self-care of patients;  - Establish individualized medical nursing programs: psychology, daily diet, disease reexamination, exercise, etc; share patients’ successful self-care: via WeChat or QQ groups, nurses provided answers to patients’ questions by specified times each week and video once a month to understand the changes in patients’ psychological emotions and communicate with them; Establishment of public account to provide various forms of health knowledge. | | 6 months;  Pretest, post-test (6 months after discharge) | | - One deputy chief physician and nurses (one chief nurse, one supervisor nurse and 4 nurses);  - Web-based (WeChat, QQ and public account). | |
| Kelleher et al (2021)  USA  [45] | Pilot RCT study | | N=31;  Had complete treatment and reported pain and psychological distress at ≥ 3;  59.5 ± 10.5;  6.4% | | - PA  - Relieve patients' pain and psychological distress through coping skills training (CST);  - Session 1-5: introduction progressive muscle relaxation; taught an activity pacing method (i.e., activity-rest cycle); taught cognitive restructuring to recognize how some thoughts can negatively influence their pain; encourage the participant to reflect on pleasant scenes and/or their own memories of such scenes; a review of all coping skills. | | 5;  45-60 minutes of each section;  3 months;  Pretest, post-test, 3 month after intervention. | | - Psychotherapist;  - Telephone;  - **Cognitive behavioral theory (focus on The Bandura’s SE theory).** | |
| Kim et al (2018)  Korea  [37] | Quasi-experimental design and comparative study | | N=118;  Had scheduled to surgery;  IG: 58 ± 10.13  CG: 63 ± 9.77;  9.92% (lost contact, death, chemotherapy) | | - PA, VP  - Improve patient quality of life, SE and anxiety and depression;  - Individual education, feedback, and support from a health professional; encourage patients to participate in mobile education via short text messages: experts through the website to check the actual learning performance of patients to complete the health goals set by themselves to enhance SE. | | 3 times telephone counseling;  2 weeks;  Pretest, post-test. | | - No specific report;  - Web-based,  telephone;  - **The** **Bandura’s SE theory.** | |
| Lim et al (2019)  Singapore  [36] | RCT | | N=51;  Had scheduled to surgery;  63 ± 13.2;  3.7% (death, dropped out) | | - PA, VP  - Improve the emotion of patients after stoma sugary, to better adapt to the stoma, and improve the QOL;  - Provide an educational booklet: introduction to psychosocial interventions, self-care strategies, community resources and support services, Stoma care strategy; guide in stoma care, stoma education protocol; Patients will learn and carry out return demonstrations to the stoma nurse specialist. The nurse will solve the patient's problems at any time through the telephone follow-up in time. | | 6;  60-minute of session, 15-minute of each telephone;  4 weeks;  Baseline, day of discharge , one and 4 months on discharge. | | - Nurse (colorectal nurse specialists);  - F2F, booklet, telephone;  - **The** **Bandura’s SE theory; the stoma acceptance conceptual framework by Simmons.** | |
| Luo et al  (2021)  China  [32] | Feasibility study (pre-post design) | | N=20 couple dyads;  Any stage;  CRC patients and spousal caregivers;  16.7% (change hospital, schedule conflict and died/too ill) | | - PA, VP  - Combined F2F and online to support CRC patients and their spousal caregivers coping with cancer together;  - Online platform consisted of 6 modules: Dyadic Learning Sessions, Health Information, Cancer News, Online Support, Sharing Circle and Personal Centre F2F consisted 5 psycho-educational sessions: take care of your spouse with cancer; Adapt to your role as patient/ caregiver; mutual support and coping together; effective and genuine communication; and rebuild confidence and return to society. | | 3 (two-, four- and six-week);  60-90 minutes each F2F section;  6 weeks;  Pretest, post test | | - Nurse (nursing graduate);  - Web-based, F2F. | |
| Reese et al (2014)  USA  [44] | Pilot RCT study | | N=23 couple dyads;  Stage I-IV;  CRC patients and spousal caregivers;  52.6 ± 10.6; | | - PA, VP  - The main goal is to reduce sexual problems;  - Teaching behavioral skills for coping with sexual challenges; improving both physical and emotional intimacy including techniques from both sex therapy and couple/marital therapy such as sensual touching exercises (i.e., sensate focus), effective sexual communication skill, and problem solving exchange to devise new intimacy activities. | | 4;  50-minute weekly;  4 weeks | | - No specific report;  - Telephone. | |
| Shepherd et al (2019)  UK  [38] | RCT | | N=132;  Stage II-IV;  IG: 62.71 ± 11.35  CG: 61.5 ± 11.99;  7.29% (no further treatment or died) | | - VP  - The aim of intervention is to improve the patient's decision-making SE;  - Accompanied IG participants to up to three appointments over a 6‐month period: (a)the first appointment in which chemotherapy as an option is discussed and planned; (b)a review of the ongoing treatment; (c)a review following the end of first line treatment. | | 3;  Pre-post first medical appointment, post second medical appointment, post third medical appointment, 3 months after clinic discharge | | - Psychotherapists;  - Telephone. | |
| Teo et al  (2020)  Singapore  [35] | RCT | | N=60;  advanced  CRC patient;  60.9 ± 9.1;  12% (progressing  illness or death) | | - PA, EA  - Explore cognitive behavioral therapy (CBT)-based intervention to improve coping with the disease;  - CBT Session 1-4: a combination of psycho-education on self-management of chronic illness and relaxation training; behavioral skills training for symptom management of common symptoms; cognitive strategies to manage worries; strategies to maximize social support and changed dynamics within the family. | | 4;  one hour of each section;  2 months;  Pretest, post-test (baseline and 8 weeks) | | - Psychotherapists;  - No report. | |
| Xu et al  (2018)  China  [31] | RCT | | N=48;  Any stage;  IG: 60.9 ± 11.47  CG:61.14 ± 13.58 | | - PA, VE, VP  - Improve patients’ SE levels and QOL;  - SE intervention: direct experience, alternative experience, verbal persuasion, social and psychological support, and adjustment of the intervention measures according to the patients’ feedback. | | 7;  3 months;  10 days, 1 month, and 3 months post-operatively. | | - Nurse;  - F2F or telephone;  - **The Bandura’s SE theory.** | |
| Zhang et al (2013)  China  [25] | RCT | | N=103;  Any stage;  IG: 52.9 ± 13.3  CG: 55.3 ± 13.7;  13.5% (death, refused because of extreme weakness, lost contact ) | | - PA, VE, VP, EA  - Improve patient ostomy adjustment level;  - Self-care skills accomplishment; vicarious experiences; verbal persuasion; emotional arousal. | | 2-3;  1 month;  Baseline (before discharge), 1 and 3 months after discharge | | - Nurse (stoma specialist nurses);  - Telephone;  - **The** **Bandura’s SE theory.** | |
| Zhang et al (2014)  China  [24] | RCT | | N=152;  Stage II-III and had scheduled to receive chemotherapy;  IG: 53.6 ± 11.3  CG: 53 ± 11.3;  20.39% (discontinued chemotherapy, not complete questionnaire) | | - PA, VE, VP, EA  - Improve SE to reduce symptom distress;  - F2F: identifying individual symptom self-management needs and strategies; booklet was used to complement the F2F education; health-coaching telephone: strengthen participants’ SE in symptom management (discussion of symptom distress, chemotherapy adherence and self-management strategies, encouragement and reinforcement to the participants’ efforts and successes, and empowered them through support; audiotape: relaxation techniques such as deep breathing and muscle relaxation. | | 5;  One-hour of F2F session and 20-40 minute of each telephone session;  6 months; | | - Nurse (four oncology nurses);  - F2F, telephone, booklet;  - **The** **Bandura’s SE theory.** | |

Abbreviations: CRC: colorectal cancer; CG: control group; F2F: face-to-face; IG: intervention group; QOL: quality of life; RCT: randomized controlled trails; SD: Study design; SE: self-efficacy.

**Intervention coverage domains^†^**: covering sources of SE (PA: Performance accomplishments; VE: vicarious experience; VP: verbal persuasion; EA: emotional arousal.

**Table S2. SE intervention outcomes**

| **Author**  **Reference number** | **Outcome measurements** | **Study outcomes (p=statistical significance * <.05; **<.01**  **Cohen’s d: between group comparisons)** | **QR** |
| --- | --- | --- | --- |
| Anderson et al. (2010)  UK  [39] | - **SE**: SE scales for diet; SE scales for physical activity;  - **QOL**: patient-generated quality-of-life questionnaires (PGI);  - **Other outcomes measurements**:  Bodily function: the Dietary Intake and Nutritional Education dietary intake (DINE); physical activity record diaries Plasma and platelet ascorbic acid measures;  In-depth interviews: assess programme acceptability. | - SE (diet and physical)***** (95%CI, 1.3, 26; p = 0.032);  - PGI showed that 14 of the 17 participants’ overall QOL had improved****** (95%CI, 17.2, 61.7, p = 0.002) and moderate activity increased at mid-study****** (95%CI, 16, 65, p = 0.003);  - DINE showed a decrease of saturated fat intake****** (95%CI, 6.5, 23.1; p = 0.002); Many participants reported overall health benefits derived from the intervention specific weight loss, increased energy and fitness, decreased constipation and improved sleeping patterns. | M |
| Bains et al. (2011)  UK  [40] | - **SE**: the Chronic Disease SE; a return-to-work SE scale;  - **Other outcomes measurements**:  Bodily function: work ability and adjustments: 3 item taken from the Work Ability Index and a list of work adjustments (reduced hours, amended tasks); Cancer related information: self-designed questionnaire to gathering information of treatment(s) (surgery, chemotherapy, radiotherapy, and/or other), prior experiences of cancer, along with any additional chronic illnesses;  Psychological distress: HADS. | Comparisons to baseline scores:  - Participants’ SE in relation to managing both cancer (*d* = 0.34) and work (*d* = 0.08) improved;  - Anxiety (*d* = 0.68) and depression (*d* = 0.61) scores decreased over time; mean perceived work ability scores showed subtle increments (*d* = 0.34). | M |
| Cramer et al. (2016)  Germany  [42] | - **SE**: the Body-Efficacy Expectation Scale;  - **QOL**: the Functional Assessment of Cancer Therapy - Colorectal (FACT-C);  - **Other outcomes measurements**:  Bodily function: Sleep disturbance the Pittsburgh Sleep Quality Inventory (PSQI); fatigue: the Functional Assessment of Chronic Illness Therapy (fatigue where higher scores indicate lower fatigue);  Psychological distress: HADS; Body awareness and bodily dissociation: the Scale of Body Connection. | - QOL (emotional well-being)* (*d* = 0.66) increased in 22 weeks;  - Less sleep disturbance* (22 weeks) (*d* = 0.34) and less anxiety* (*d* = 0.65) and depression* (*d* = 0.59) (10 weeks). | M |
| Döking et al. (2021)  Netherlands  [43] | - **SE**: The SE Scale-28 (SE-28);  - **QOL**: the European Organization for Research and Treatment (EORTC) of Cancer Quality of Life Questionnaire-Core 30 (QLQ-C30+CR38);  - **Other outcomes measurements**:  Bodily function: fatigue: the Checklist Individual Strength (CIS);  Psychological distress: Distress: the Impact of Event Scale (IES); HADS; the Brief Symptom Inventory-18 (BSI-18); Fear of cancer recurrence: the Cancer Worry Scale (CWS);  Program evaluation: Therapeutic relationship: the short version of the Working Alliance Inventory (WAI-S); Treatment satisfaction, efficacy, and user-friendliness: an intervention evaluation Questionnaire;  The evaluation of the therapeutic alliance, the effectiveness of and satisfaction with the intervention, and the intervention design: semi-structured interview. | - SE: SE-28 scores increased during T4 (RCI = 3.21);  - QOL: emotional between T1 and all measurement times (RCI_T1-T2_ = 4.19, RCI_T1-T3_ = 3.35, RCI_T1-T4_ = 3.35), social function in T1-T2 (RCI = 2.35), global QOL in T2-T3 (RCI_T1-T2_ = 2.86, RCI_T1-T3_ = 2.14), body image (RCI = 2.36), sexual enjoyment (RCI = 2.42), future perspective (RCI = 2.18);  **- A lower score indicates better outcomes**: BSI total T1-T2  (RCI = -2.23), depression T1-T2 (RCI = -2.19); anxiety T1 and all other assessments; HADS total and anxiety in T2 and both follow-ups (RCI = -2.66); CIS fatigue in T1-T3 (RCI = -2.03); IES both follow-up (RCIT1-T3 = -5.47, RCIT1-T4 = -3.65); pain (RCI = -3.61), insomnia (RCI = -2.20), stoma-related problems (RCI = -2.47) in T1-T2. | M |
| Gao et al. (2017)  China  [30] | - **SE**^†^: The Stoma SE Scale (SSES);  - **Other outcomes measurements**:  Bodily function: Self-care ability scale;  Complication: check and judge by 2 Stoma team members. | - IG SE higher than CG after 6 months**;  - IG self-care ability, self-concept, health knowledge level, self-care skills and sense of self-responsibility have been significantly improved after intervention*; and has lower complication rate than CG*. | W |
| Gao et al. (2017)  China  [34] | - **SE**: Colostomy self-care manage management questionnaire (for stages of change, decisional balance, processes of change, and SE);  - **QOL**: QOL scale (no specific report). | - After 1 month and 6 months, IG SE (*d* = 0.77-1.79), overall QOL (*d* = 2.35-3.63,), and other 4 self-care management variables were all higher than CG******. | W |
| Giesler et al. (2017)  Germany  [41] | - **SE**^†^: the Cancer Behavior Inventory (CBI-B-D) to evaluate SE for coping with cancer;  - **Other outcomes measurements**:  Bodily function: self-rating measure of patient competence (PEPK 2-57). | - No positive results (SE less than baseline at T2,T3 [2, 6 weeks]; patient competence less than T1 (baseline) at T2 ,T3 [2,6 weeks]). | M |
| Huang et al. (2021)  China  [33] | - **SE**: The General SE Scale (GSES);  **- QOL**: 36-Item Short Form Health Survey (SF-36);  - **Other outcomes measurements**:  Bodily function: the Self-care Ability Scale (ESCA);  Psychological distress: the Self-rating Anxiety Scale (SAS) and the Self-rating Depression Scale (SDS). | - SE increased** (*d* = 1.07), all of QOL variables** (*d* = 0.95-1.66), SAS** (*d* = 0.78), SDS** (*d* = 0.90), and all of self-care ability variables* (*d* > 0.86). | S |
| Kelleher et al (2021)  USA  [45] | - **SE**: the SE for Pain Management subscale of the Chronic Pain SE Scale;  - **QOL**: the 27-item Functional Assessment of Cancer Therapy-General (FACT-G);  - **Other outcomes measurements**:  Bodily function: the 4-item Pain Severity sub scale of the Brief Pain Inventory (BPI);  Psychological distress: the 18-item Brief Symptom Inventory (BSI-18). | - Post-treatment: SE (*d* = 0.21), BPI (*d* = 0.5), psychological distress (*d* = 0.20) improved;  - 3-month: SE (*d* = 0.27), overall QOL (*d* = 0.40), BPI (*d* = 0.5), psychological distress (*d* = 0.17) improved. | M |
| Kim et al (2018)  Korea  [37] | - **SE**: Korean version of the Hospital Anxiety Depression Scale;  - **QOL**: the Functional Assessment of CRC survey (FACT-Cv4);  - **Other outcomes measurements**:  Psychological distress: HADS. | - SE** (*d* = 0.37), QOL (affective status) increased** (*d* = 0.57);  - Anxiety** (*d* = 0.69) and depression** (*d* = 0.84) decreased. | M |
| Lim et al (2019)  Singapore  [36] | - **SE**^†^: the Stoma SE Scale (SSES);  - **QOL**: the European Organization for Research and Treatment of Cancer Quality of Life Questionnaire Colorectal 29-item questionnaire (EORTC QLQ-CR29);  - **Other outcomes measurements**:  Bodily function: the Acceptance of Chronic Health Conditions Scale (ACHC);  Psychological distress: HADS. | - Anxiety decreased in 1 (*d* = 0.65) and 4-month follow-up**  (*d* = 0.77); acceptance of stoma increased in 1-month follow-up** (*d* = 0.82) and 4-month follow-up** (*d* = 0.82). | M |
| Luo et al  (2021)  China  [32] | - **SE**^†^: the 12-item Cancer Behavior Inventory (CBI-B);  - **QOL**: the medical outcomes study 12-item short form (SF-12) (physical and mental);  - **Other outcomes measurements**:  Psychological distress: the 37-item Dyadic coping Inventory (DCI); HADS; 17-item benefit-finding scale (BFS-C);  Dyadic relationship: 15-item Cancer-Related Communication Problems within Couples Scale (CRCP); 14-item revised dyadic adjustments scale (RDAS). | Comparisons to baseline scores:  - SE: small-to-medium improvements in CRC patients (*d* = 0.36) and spousal caregivers (*d* = 0.37);  - Patients: positive effect found in SF-12 (*d* = 0.18, *d* = 0.18); DCI (*d* = 0.25); RDAS (*d* = 0.16); HADS (anxiety: *d* = 0.12, depression: *d* = 0.14); BFS-C (*d* = 0.27-0.65);  - Spousal caregivers: positive effect found in SF-12 (*d* = -0.004,  *d* = 0.33); DCI (*d* = 0.21); RDAS (*d* = 0.10); HADS (anxiety:  *d* = 0.16, depression: *d* = 0.37); BFS-C (*d* = 0.14-0.27). | M |
| Reese et al (2014)  USA  [44] | - **SE**: Sex-related SE questionnaire;  - **Other outcomes measurements**:  Bodily function: the total sexual function scores from the Female Sexual Function Index; the Medical Impact subscale of the Sexual Function Questionnaire;  Psychological distress: the Index of Sexual Satisfaction;  Dyadic relationship: the 13-item Dyadic Sexual Communication Scale; the Miller Social Intimacy Scale. | - SE: have found improvement in CRC patients (enjoying intimacy, *d* = 0.66) and spousal caregivers (*d* ≧ 0.57).  - Patients: positive effect found in female and male sexual function (*d* = 0.85 and *d* = 0.58, respectively), and medical impact on sexual function (*d* = 0.66);  - Spousal caregivers: positive effect found in female and male sexual function (*d* = 0.18 and *d* = 1.76,respectively) , and medical impact on sexual function (*d* = 0.32), sexual distress (*d* = 0.69), communication (*d* = 0.97), intimacy (*d* = 0.51). | M |
| Shepherd et al (2019)  UK  [38] | - **SE^†^**: the Decision Self‐Efficacy scale (DSE);  - **Other outcomes measurements**:  Psychological distress: HADS; Perception of the decision made: Decisional Conflict Scale (throughout treatment) (DCS); the Decision Regret Scale( the end of treatment) (DRS);  Assess the intervention: four items were selected, based on CPRS evaluation from the Preparation for Decision-Making scale (PfDM). | - SE: The consultation intervention before the first visit* (*d* = 0.44) and the third consultation intervention** (*d* = 0.72);  **- A lower score indicates better outcomes**: less depression**, decision conflict (post second medical consultation)**  (*d* = 0.59)and regret (3-month follow-up)* (*d* = 0.54). | M |
| Teo et al  (2020)  Singapore  [35] | - **SE**: the Cancer Behavior Inventory, Version 2 (three subscales) ;  - **Other outcomes measurements**:  Psychological distress: HADS. | - Post-treatment (8 week): SE increased (use of information seeking (*d* = 0.21), coping with side effects (*d* = 0.10), acceptance/positive attitude (*d* = 0.21));  - Distress score decreased (*d* = 0.05). | M |
| Xu et al  (2018)  China  [31] | - **SE^†^**: the Stoma SE Scale (SSES);  - **QOL**: The European Organization for Research and Treatment of Cancer Quality of Life Questionnaire Cancer 30 (EORTC QLQ-C30 V3.0). | - SE increased in1-month follow-up* (*d* = 0.59) and 3-month follow-up** (*d* = 2.41);  - 12 of the 15 variables that measure QOL have been significantly improved (except dyspnea, constipation and economic difficulties) (*d* = 0.16 - 0.95). | W |
| Zhang et al (2013)  China  [25] | - **SE**: the Stoma SE Scale (SSES);  - **Other outcomes measurements**:  Bodily function: the Ostomy Adjustment Scale (OAS)  Satisfaction with care: a single self-reported item;  Stoma complications: identified by an enterostomal nurse according to a preset checklist. | - SE increased in 1-month follow-up** (*d* = 0.21) and 3-month follow-up** (*d* = 0.49);  - Ostomy adjustment increased in**1-month follow-up** (*d* = 0.34) and 3-month follow-up** (*d* = 0.55); less stoma complications*, high satisfaction** in 1- and 3-month follow-up. | M |
| Zhang et al (2014)  China  [24] | - **SE^†^**: the Stanford Inventory of Cancer Patient Adjustment (SICPA);  - **QOL**: the Functional Assessment of Cancer Treatment-G (FACT-G, Version 4);  - **Other outcomes measurements**:  Bodily function: the M.D. Anderson Symptom Inventory (MDASI);  Psychological distress: HADS. | SE’s 7 variables were assessed and resulting in 13 effect sizes for 3-month follow-up and 6-month follow-up:  - 3-month: total* (*d* = 0.53); coping (*d* = 0.50); communication*  (*d* = 0.51); activity management** (*d* = 0.52); personal management* (*d* = 0.44); affective management* (*d* = 0.44);  - 6-month: total* (*d* = 0.88); coping (*d* = 0.55); communication*  (*d* = 0.95) activity management** (*d* = 0.79); personal management* (*d* = 0.65); affective management* (*d* = 0.77); self-satisfaction (*d* = 0.11). | S |

Abbreviations: 95%CI: 95% confidence interval; CRC: colorectal cancer; CG: control group; HADS: the Hospital Anxiety Depression Scale; IG: intervention group; QOL: quality of life; QR: Quality Rating; RCI: Reliable Change Index, the change is considered reliable when RCI has a greater value than 1.96 (p < .05); SD: Study design; SE: self-efficacy; ^†^: primary outcome.
